# Supplementary material for: Persistent Immunity against SARS-CoV-2 in Individuals with Oncohematological Diseases Who Underwent Autologous or Allogeneic Stem Cell Transplantation after Vaccination
Source: Cancers (Basel). 2023 Apr 18;15(8):2344. doi: 10.3390/cancers15082344 (PMC10137176; doi:10.3390/cancers15082344)
Supplement: Supplementary file 1 [file cancers-15-02344-s001.zip › Suplementary/Table S2.docx]

**Table S2.** Sociodemographic and clinical data of healthy donors who participated in the study as controls.

| **Participant´s code** | **Age (years)** | **Gender (M/F)** | **Comorbidities** | **Type of COVID-19 vaccine** | **No. of doses received to complete vaccination schedule** | **Time between doses (days)** | **Time from complete vaccination schedule to 1^st^ sample (days)** | **Time from complete vaccination schedule to 2^nd^ sample (days)** | **Time from 1^st^ to 2^nd^ sample (days)** | **SARS-CoV-2 breakthrough infection**  **(Y/N; Severity)** |
| --- | --- | --- | --- | --- | --- | --- | --- | --- | --- | --- |
| CTV_16 | 71 | M | None | Comirnaty | 2 | 21 | 23 | 93 | 70 | N |
| CTV_24 | 71 | M | Dyslipidemia | Comirnaty | 2 | 21 | 23 | 93 | 70 | Y; Mild |
| CTV_37 | 50 | M | None | Comirnaty | 2 | 21 | 29 | 92 | 63 | N |
| CTV_38 | 48 | M | HBP, DM, obesity | Comirnaty | 2 | 21 | 24 | 87 | 63 | N |
| CTV_39 | 47 | M | Dyslipidemia | Comirnaty | 2 | 21 | 36 | 99 | 63 | N |
| CTV_41 | 40 | M | Obesity | Comirnaty | 2 | 21 | 24 | 87 | 63 | N |
| CTV_50 | 55 | F | None | Spikevax | 2 | 28 | 23 | 81 | 58 | Y; Mild |
| CTV_51 | 55 | F | Asthma | Spikevax | 2 | 28 | 30 | 88 | 58 | Y; Mild |
| CTV_52 | 45 | M | None | Comirnaty | 2 | 21 | 23 | 86 | 63 | N |
| CTV_55 | 46 | M | None | Comirnaty | 2 | 21 | 32 | 93 | 61 | N |
| CTV_59 | 53 | M | Dyslipidemia | Comirnaty | 2 | 21 | 23 | 86 | 63 | N |
| CTV_64 | 36 | M | None | Comirnaty | 2 | 21 | 37 | 95 | 58 | Y; Mild |
| CTV_65 | 44 | M | Dyslipidemia, obesity | Comirnaty | 2 | 21 | 29 | 92 | 63 | N |
| CTV_68 | 25 | F | None | Comirnaty | 2 | 21 | 22 | 85 | 63 | N |
| CTV_71 | 67 | F | Asthma | Comirnaty | 2 | 21 | 28 | 86 | 58 | Y; Mild |
| CTV_72 | 67 | M | HBP | Comirnaty | 2 | 21 | 28 | 91 | 63 | Y; Mild |
| CTV_89 | 57 | F | Obesity | Comirnaty | 2 | 21 | 29 | 94 | 65 | Y; Mild |
| CTV_99 | 39 | M | None | Comirnaty | 2 | 23 | 49 | 111 | 62 | Y; Mild |

DM, Diabetes Mellitus; F, Female; HBP, High blood pressure; M, male.
