# Supplementary material for: Persistent Immunity against SARS-CoV-2 in Individuals with Oncohematological Diseases Who Underwent Autologous or Allogeneic Stem Cell Transplantation after Vaccination
Source: Cancers (Basel). 2023 Apr 18;15(8):2344. doi: 10.3390/cancers15082344 (PMC10137176; doi:10.3390/cancers15082344)
Supplement: Supplementary file 1 [file cancers-15-02344-s001.zip › Suplementary/Figure S1.pptx]

## Slide 1
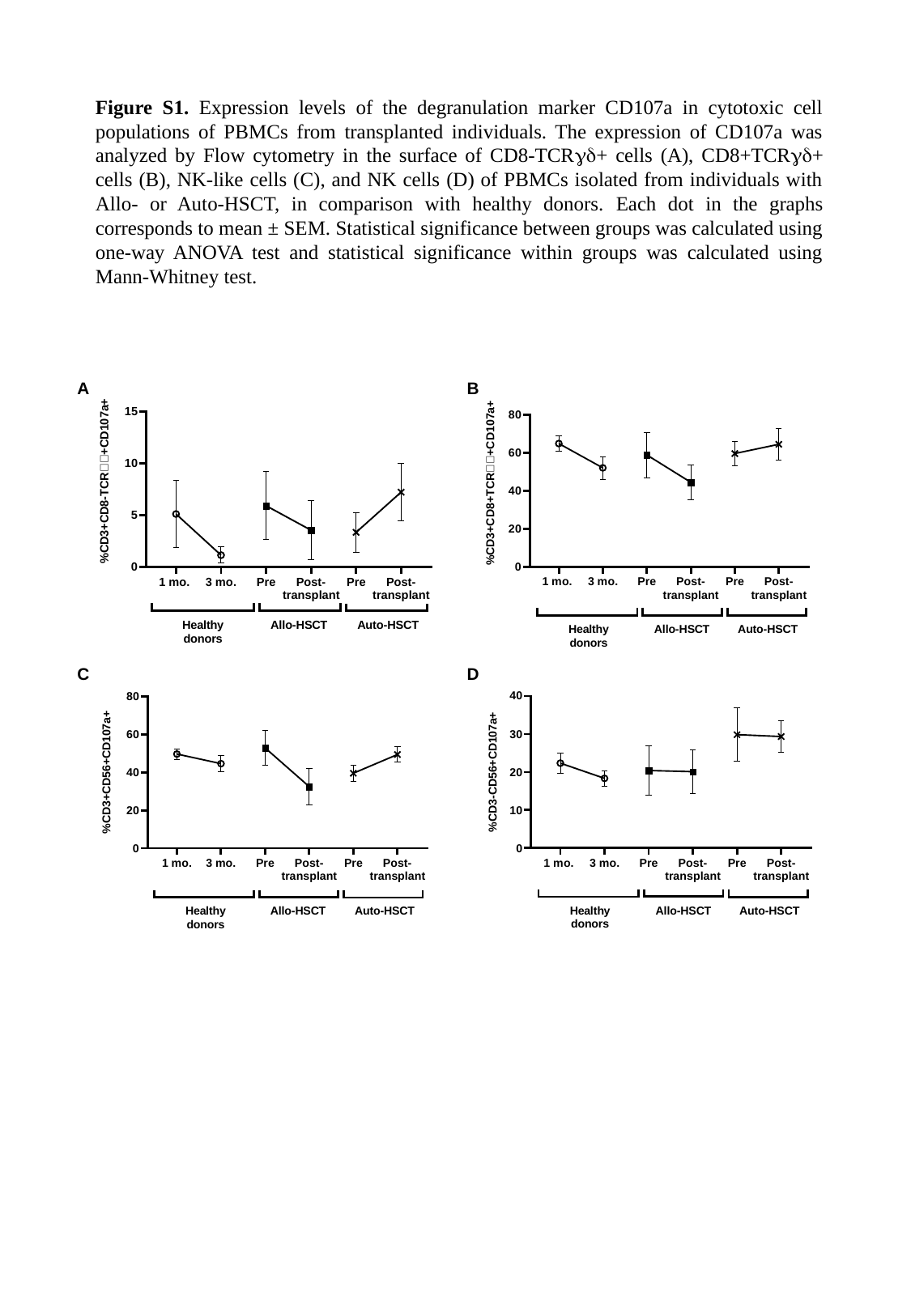

Figure S1. Expression levels of the degranulation marker CD107a in cytotoxic cell populations of PBMCs from transplanted individuals. The expression of CD107a was analyzed by Flow cytometry in the surface of CD8-TCRgd+ cells (A), CD8+TCRgd+ cells (B), NK-like cells (C), and NK cells (D) of PBMCs isolated from individuals with Allo- or Auto-HSCT, in comparison with healthy donors. Each dot in the graphs corresponds to mean ± SEM. Statistical significance between groups was calculated using one-way ANOVA test and statistical significance within groups was calculated using Mann-Whitney test.
A
B
C
D
