# Supplementary material for: Persistent Immunity against SARS-CoV-2 in Individuals with Oncohematological Diseases Who Underwent Autologous or Allogeneic Stem Cell Transplantation after Vaccination
Source: Cancers (Basel). 2023 Apr 18;15(8):2344. doi: 10.3390/cancers15082344 (PMC10137176; doi:10.3390/cancers15082344)
Supplement: Supplementary file 1 [file cancers-15-02344-s001.zip › Suplementary/Table S1.docx]

**Table S1.** Clinical data of individuals with allogenic or autologous transplant who participated in this study.

| **Patients’ code** | **Participants’ clinical data** | | | | | **Transplant clinical data** | | | | **COVID-19 vaccine** | | | | **Post-transplant parameters at the time of second sample collection** | | | | |
| --- | --- | --- | --- | --- | --- | --- | --- | --- | --- | --- | --- | --- | --- | --- | --- | --- | --- | --- |
|  | **Age**  **(years)** | **Gender**  **(M/F)** | **Underlying disease** | **Previous therapy** | **Ig deficiency** | **HSCT** | **According to the donor** | **Conditioning regimen** | **GvHD profilaxis** | **Patients vaccine type**  **(n. doses)** | **Mean time from last vaccine dose to transplant (days)** | **Donors vaccine type**  **(n. doses)** | **Donor vaccination - transplant, days** | **GvHD** | **Immunosuppression at time of second sampling** | **CMV replication** | **Disease relapse** | **Admitted to ICU** |
| TPC-10 | 51 | F | MDS | Azacitidine | No | Allo. | RD; Haploidentical | MA | Cy-Csa-MMF | Spikevax (x2) | 21 | Comirnaty (x2) | 10 | No | Csa | No | No | No |
| TPC-12 | 57 | M | MPS (MF) | Ruxolitinib | No | Allo. | RD; Haploidentical | RIC | Cy-Csa-MMF | Spikevax (x2) | 88 | Spikevax (x2) | 12 | No | Tacrolimus + MMF | No | No | No |
| TPC-16 | 58 | F | AML | IC + midostaurin | No | Allo. | RD; identical | MA | Csa-Mtx | Spikevax (x2) | 84 | Comirnaty (x2) | 62 | No | Csa | No | No | No |
| TPC-20 | 63 | M | MDS | IC | No | Allo. | RD; Haploidentical | RIC | Cy-Csa-MMF | Spikevax (x2) | 91 | Comirnaty (x2) | 15 | No | Csa + MMF + prednisone | Yes | No | No |
| TPC-21 | 65 | M | AML | IC + midostaurin | IgM, IgA deficiency | Allo. | RD; Haploidentical | RIC | Cy-Csa-MMF | Vaxzevria (x2) | 113 | Vaxzevria (x1) | 51 | No | Csa | Yes | No | Yes |
| TPC-23 | 48 | M | AML | IC + quizartinib | No | Allo. | RD; Haploidentical | MA | Cy-Csa-MMF | Spikevax (x2) | 76 | Comirnaty (x2) | 22 | No | Csa | No | No | No |
| TPC-31 | 63 | F | APL | Alentuzumab | No | Allo. | RD; Haploidentical | RIC | Cy-Csa-MMF | Spikevax (x2) | 126 | Jcovden (x1) | 69 | No | Csa + metilprednisolona | Yes | No | Yes |
| TPC-37 | 60 | M | MDS | Daunorubicin - cytarabine | No | Allo. | RD; identical | RIC | Cy-Csa-MMF | Spikevax (x3) | 147 | Comirnaty (x3) | 101 | Yes | Csa + MMF | No | Yes | No |
| TPC-42 | 68 | M | AML | IC | No | Allo. | RD; Haploidentical | RIC | Cy-Csa-MMF | Spikevax (x2) | 172 | Comirnaty (x2) | 129 | No | Csa + MMF | No | No | No |
| TPC-47 | 63 | F | ALL | IC | IgG deficiency | Allo. | RD; identical | RIC | Csa-MMF | Comirnaty (x2) | 298 | Comirnaty (x2) | 216 | Yes | Csa + prednisone | Yes | No | No |
| TPC-49 | 52 | M | CLL | Venetoclax - Rituximab | IgM, IgA, IgG deficiency | Allo. | UR; mismatch | RIC | Cy-Csa-MMF | Comirnaty (x2) | 53 | Unk. | Unk. | No | Csa | Yes | No | No |
| TPC-14 | 46 | M | MM | Cyclophosphamide-Bortezomid- Dexametasone | IgM deficiency | Auto. | NA | Melphalan 140 | NA | Spikevax (x2) | 38 | NA | NA | NA | No | NA | No | No |
| TPC-22 | 56 | M | MM | Bortezomib-Talidomide-Dexametasone | IgM, IgA deficiency | Auto. | NA | Melphalan 200 | NA | Spikevax (x2) | 27 | NA | NA | NA | No | NA | No | No |
| TPC-25 | 50 | M | NHL | DHAP | No | Auto. | NA | BEAM | NA | Spikevax (x2) | 76 | NA | NA | NA | No | NA | No | No |
| TPC-26 | 34 | M | MM | Bortezomib-Lenalidomide-Dexametasone | IgG deficiency | Auto. | NA | Melphalan 200 | NA | Spikevax (x2) | 75 | NA | NA | NA | No | NA | No | No |
| TPC-29 | 70 | F | NHL | Rituximab-CHOP | IgM, IgA deficiency | Auto. | NA | Adjusted BEAM | NA | Spikevax (x2) | 94 | NA | NA | NA | No | NA | No | No |
| TPC-30 | 43 | M | NHL | RItuximab-ESHAP | IgM, IgA, IgG deficiency | Auto. | NA | BEAM | NA | Spikevax (x2) | 106 | NA | NA | NA | No | NA | No | No |
| TPC-32 | 52 | M | MM | Daratumumab-Lenalidomide-Dexametasone | IgM, IgA, IgG deficiency | Auto. | NA | Melphalan 200 | NA | Spikevax (x2) | 125 | NA | NA | NA | No | NA | No | No |
| TPC-33 | 60 | F | MM | Bortezomib-Lenalidomide-Dexametasone | IgG deficiency | Auto. | NA | Melphalan 200 | NA | Spikevax (x2) | 119 | NA | NA | NA | No | NA | No | No |
| TPC-35 | 65 | M | MM | Bortezomib-Lenalidomide-Dexametasone | IgM, IgG deficiency | Auto. | NA | Melphalan 200 | NA | Vaxzevria (x2) | 100 | NA | NA | NA | No | NA | No | No |
| TPC-36 | 63 | M | NHL | Rituximab-ESHAP | IgM, IgA deficiency | Auto. | NA | Adjusted BEAM | NA | Spikevax (x2) | 133 | NA | NA | NA | No | NA | No | No |
| TPC-38 | 27 | F | HL | Brentuximab-ESHAP | No | Auto. | NA | BEAM | NA | Vaxzevria (x2) | 239 | NA | NA | NA | No | NA | No | No |
| TPC-39 | 64 | F | MM | Bortezomib-Lenalidomide-Dexametasone | IgM, IgA, IgG deficiency | Auto. | NA | Melphalan 140 | NA | Vaxzevria (x2) | 125 | NA | NA | NA | No | NA | No | No |
| TPC-41 | 69 | M | MM | Bortezomib-Lenalidomide-Dexametasone | IgM, IgA, IgG deficiency | Auto. | NA | Melphalan 140 | NA | Vaxzevria (x2) | 131 | NA | NA | NA | No | NA | No | No |
| TPC-43 | 59 | M | MM | Bortezomib-Talidomide-Dexametasone | IgM deficiency | Auto. | NA | Melphalan 200 | NA | Comirnaty (x2) | 152 | NA | NA | NA | No | NA | No | No |
| TPC-45 | 45 | M | NHL | Rituximab-CHOP/ Rituximab-DHAP | IgM, IgA deficiency | Auto. | NA | BEAM | NA | Spikevax (x2) | 164 | NA | NA | NA | No | NA | No | No |
| TPC-46 | 66 | M | MM | Bortezomib-Talidomide-Dexametasone.  Lenalidomide – dexametasone. | IgM deficiency | Auto. | NA | Melphalan 200 | NA | Comirnaty (x2) | 248 | NA | NA | NA | No | NA | No | No |
| TPC-50 | 65 | F | NHL | Rituximab-ESHAP | IgM, IgA deficiency | Auto. | NA | Adjusted BEAM | NA | Spikevax (x3) | 153 | NA | NA | NA | No | NA | No | No |
| TPC-51 | 52 | M | NHL | Rituximab-CHOP/ Rituximab-DHAP | No | Auto. | NA | BEAM | NA | Comirnaty (x2) | 205 | NA | NA | NA | No | NA | No | No |

Allo., allogenic; AML, acute myeloid leukemia; ALL, acute lymphocytic leukemia; APL, acute prolymphocytic leukemia; Auto., Autologous; BEAM, carmustine (BCNU), etoposide (ETP), cytarabine (Ara‐C), and melphalan; CHOP, Cyclophosphamide-Doxorubicin-Vincristine-Prednisolone; CLL, chronic lymphocytic leukemia; CMV, citomegalovirus; Csa, cyclosporine; Cy, cyclophosphamide; DHAP, Dexamethasone-High-dose Cytarabine (Ara-C)-Cisplatin; ESHAP, Etoposide-Methylprednisolone- High-dose Cytarabine (Ara-C)-Cisplatin; GvHD, graft-versus-host-disease; HL, Hodgkin’s lymphoma; HSCT, haematopoietic stem cell transplantation; IC, intensive chemotherapy; ICU, intensive care unit; MA, myeloablative; MDS, myelodysplastic syndrome; MF, myelofibrosis; MM, multiple myeloma; MMF, mycophenolate mofetil; MPS, myeloproliferative syndrome; Mtx, methotrexate; NA, not applicable; NHL, Non-hodgkin’s lymphoma; RIC, reduced-intensity conditioning; RD, related donor; UD, unrelated donor; Unk., Unknown.
